# Sensitization to common aeroallergens in a population of young adults in a sub-Saharan Africa setting: a cross-sectional study

**DOI:** 10.1186/s13223-015-0107-8

**Published:** 2016-01-04

**Authors:** Bertrand Hugo Mbatchou Ngahane, Diane Noah, Malea Nganda Motto, Yacouba Mapoure Njankouo, Louis Richard Njock

**Affiliations:** Department of Internal Medicine, Douala General Hospital, PO Box 4856, Douala, Cameroon; Faculty of Medicine and Pharmaceutical Sciences, University of Douala, Douala, Cameroon; Department of Ear Nose and Throat, Douala General Hospital, Douala, Cameroon; Faculty of Medicine and Biomedical Sciences, University of Yaounde 1, Yaounde, Cameroon

**Keywords:** Allergic sensitization, Skin prick test, Asthma, Allergic rhinitis, Africa

## Abstract

**Background:**

Sensitization to aeroallergens increases the risk of developing asthma or allergic rhinitis. Data on sensitization to airborne allergens in the general population in sub-Saharan Africa are lacking. The aim of this study was to determine the prevalence and determinants of sensitization to common aeroallergens in a population of young adults.

**Methods:**

A cross-sectional study was conducted among students of the Faculty of Medicine and Pharmaceutical Sciences of the University of Douala between 1st February and 30th April 2014. We consecutively recruited all the students present in class or in hospital during our visit. They filled an anonymous questionnaire and underwent skin prick tests with common aeroallergens. A logistic regression model of the SPSS.20 software was used to investigate factors associated with sensitization to common aeroallergens.

**Results:**

Of the 600 students included in the study, 305 (50.8 %) were female. The mean age of participants was 22.6 ± 2.7 years. The prevalence of sensitization to aeroallergens was 42.8 % (95 % CI 38.8–46.8). *Dermatophagoides pteronyssimus* (24.2 %), *Dermatophagoides farinae* (22.8 %), *Blomia tropicalis* (23.3 %) and *Blatella germanica* (15.2 %) were the most common allergens found. Allergic rhinitis, asthma symptoms and family atopy were independently associated to sensitization to common aeroallergens.

**Conclusion:**

A significant proportion of young adults are sensitized to common aeroallergens. Dust mites and cockroach should be included in the panel of aeroallergens in Cameroon.

## Background

The prevalence of allergic diseases is steadily increasing especially in middle- and low-income countries. The main manifestations of respiratory allergy which are asthma and allergic rhinitis are increasing rapidly these last 2 decades [1]. The number of asthma and allergic rhinitis patients worldwide are estimated at 334 and 400 million respectively [1, 2]. Asthma is associated with allergic rhinitis in 74–81 % of cases [3] and is one of highest ranking specific diseases in terms of years lost to disability adjusted life years [4]. Atopy or allergic sensitization is defined as the production of immunoglobulin E (IgE) in response to allergens, especially inhaled allergens and food allergens [1]. It is an important step in the pathogenesis of IgE mediated allergic diseases and is therefore a major risk factor for the development of asthma and allergic rhinitis [1, 5, 6]. In order to design efficient preventive measures, it is useful to assess the prevalence and risk factors of allergic sensitization.

In industrialized countries, there has been an increase over time of the sensitization to aeroallergens in the general population with a prevalence ranging from 25 to 50 % [7, 8]. In Africa, most studies have been carried out in selected populations of asthma patients or allergic rhinitis patients. Nevertheless, Benzarti et al. in Tunisia found a 34 % sensitization to common aeroallergens in a population of 500 subjects of an unselected population [9]. In another study carried out in Uganda, 14 and 20 % of participants were respectively sensitized to *Blomia tropicalis* (BP) and *Dermatophagoides pteronyssinus* [10]. In Cameroon, we could not identify any studies on sensitization to aeroallergens in the general population. The objective of this study was to determine the prevalence and factors associated with sensitization to common allergens in a population of young adults.

## Methods

### Design and study setting

We conducted a cross-sectional study from February 1st to April 30th, 2014 among students of the Faculty of Medicine and Pharmaceutical Sciences of the University of Douala, who represented a sub-set of young adults in Douala, the most populated city of Cameroon. This seaside city is characterized by an equatorial climate with temperatures ranging from 21 to 31 °C with high rainfall from June to October. The relative humidity varies between 80 and 90 % over the year.

### Participants

The study population was made of students of the Faculty of Medicine and Pharmaceutical Sciences. We included students attending classes and those present in the hospitals for their internship during the study period. Pregnant students, those on antihistamines, those presenting with acute asthma and non consenting students were excluded. Based on an expected prevalence of sensitization to common aeroallergens of 34 % [9] and considering an accepted error of 5 % and a 95 % confidence interval, a minimal sample size of 268 participants was required for this study.

### Data collection

The recruitment of participants was completed at the university campus during break periods for students from grade 1 to grade 5 and in hospitals for grade 6 and grade 7 students. Using a self-administered questionnaire, the following data were collected: socio-demographic characteristics, home environment, smoking habits, personal and family history of atopy and asthma and allergic rhinitis symptoms. The questionnaires were returned to the investigator.

Skin prick tests were performed by two trained investigators according to the recommendations of the European Academy of Allergy and Clinical Immunology [11]. We used 8 standardized allergen extracts provided by ALK laboratories (Argonne in Varennes, France): the house dust mites *Dermatophagoides pteronyssinus, Dermatophagoides farina*e and *Blomia tropicalis*, the moulds *Alternaria alternata* and *Aspergillus fumigates*, cat dander, dog dander, and the cockroach *Blatella germanica.* Glycerosaline solution was used as the negative control and Histamine dihydrochloride 10 mg/ml as the positive control.

After cleaning of the volar aspect of the forearm with alcohol, a single drop of each test solution was applied on the skin. A skin-prick test was performed within the drop by pressing a lancet through the drop of allergen extract and holding it against the skin for at least 1 s. The results of the tests were read 15 min following the application by measuring the largest diameter of the wheal of each allergen. The test was considered positive when the wheal diameter was greater or equal to 3 mm. Allergic sensitization was defined as at least one positive reaction to the 8 aeroallergens used.

### Statistical analysis

Data were entered and analyzed using IBM SPSS Statistics version 20.0. (Armonk, NY, USA: IBM Corp). Quantitative variables were summarized as means and standard deviations and qualitative data as frequencies and percentages. Univariate and multivariate logistic regression was used to determine the association between independent variables and sensitization to common aeroallergens. Variables with p < 0.2 in the univariate analysis were fitted into the final multiple logistic regression models. Variables with p < 0.05 in the final model were taken as significant associated factors.

### Ethical clearance

The study protocol was given ethical approval by the ethics committee of the Douala University and verbal consent was obtained from each participant before recruitment.

## Results

Of the total of 680 students who received questionnaires, 608 returned the questionnaires to the investigator and underwent skin prick tests, giving a response rate of 89.4 %. Prick tests from eight subjects were not interpretable. The final study population consisted of 600 students of which 305 (58.8 %) were female. The mean age of participants was 22.7 ± 2.8 years (range 16–35). Symptoms suggestive of asthma and allergic rhinitis were found in 50 (8 %) and 301 (50.2 %) participants, respectively. The other characteristics of the study population are illustrated in Table 1.

**Table 1:** Baseline characteristics of participants (N = 600)

Variables	Number	Percentage
Gender		
Male	295	49.2
Female	305	50.8
Age (years)		
<20	79	13.2
20–24	359	59.8
25–29	158	26.3
≥30	4	0.7
Family atopy		
Yes	221	36.8
No	379	63.2
Diagnosed asthma		
Yes	39	6.5
No	561	93.5
Allergic rhinitis		
Yes	87	14.5
No	513	85.5
Atopic dermatitis		
Yes	51	8.5
No	549	91.5
Allergic conjunctivitis		
Yes	37	6.2
No	563	93.8
Smoking		
Yes	11	1.8
No	589	98.2
Alcohol consumption		
Yes	46	7.7
No	554	92.3
Moulds		
Yes	352	58.7
No	248	41.3
Carpet at home		
Yes	397	66.2
No	203	33.8
Allergic rhinitis symptoms		
Yes	301	50.2
No	299	49.8
Wheezing the last 12 months		
Yes	50	8.3
No	550	91.7

**Fig. 1:**
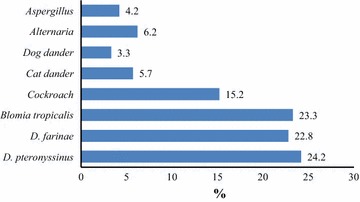
Prevalence of sensitization to aeroallergens

**Fig. 2:**
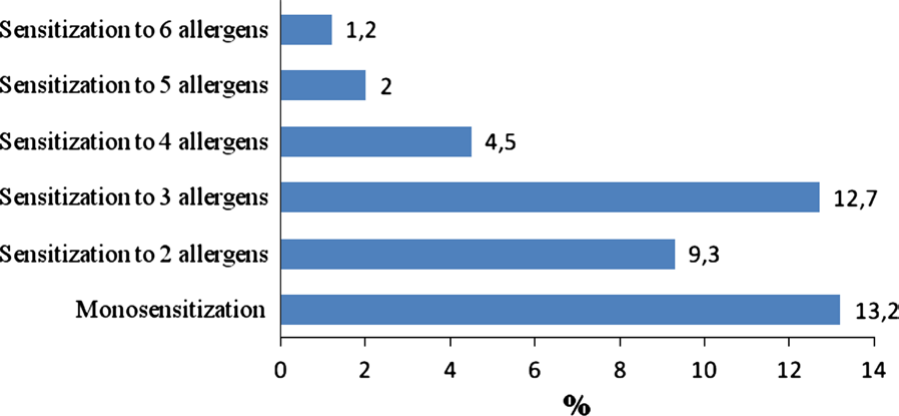
Sensitization to one or more aeroallergens

The skin prick tests were positive for at least one allergen in 257 subjects, giving a prevalence of allergic sensitization of 42.8 % (95 % CI 38.8–46.8). *D. farinae* (24.2 %), *B. tropicalis* (23.3 %), *D. pteronyssinus* (22.8 %) and cockroach (15.2 %) were the most prevalent sensitizers (Fig. 1). The skin prick tests were positive for the 3 mites in 87 (14.5 %) participants while positive to both *D. farinae* and *D. pteronyssinus* in 107 (17.8 %) participants. The reactions were negative for 57.2 % of participants. Sensitization to only one allergen was present in 79 (13.2 %) participants while the sensitization to 4 different allergens was observed in 4.5 % of participants (Fig. 2). Of the 79 subjects with a monosensitization, cockroach was most represented (36, 7 %) followed by *D. pteronyssinus* (19 %) and B*. tropicalis* (13.9 %).

Univariate analysis indentified personal history of atopic dermatitis, family atopy, being exposed to cat at home, allergic rhinitis and wheezing during the 12 last months were significantly associated with sensitization to any aeroallergen (Table 2). The multivariate analysis revealed that, family atopy (OR 1.47, 95 % CI 1–2.1), wheezing during the last 12 months (OR 3.12, 95 % CI 2.19–4, 42) and allergic rhinitis symptoms (2.43, 95 % CI 1.27–4.66) were the factors independently associated to sensitization with common aeroallergens.

**Table 2:** Univariate and multivariate analysis of factors associated with sensitization to aeroallergens

Variable	Positive skin prick test	P value	Adjusted odds ratio	Adjusted P value
Age (increase in)		0.15	1.06 (0.99–1.13)	0.06
BMI (increase in)		0.46	–	–
Sex				
Male	121 (41 %)	0.77	–	–
Female	135 (44.6 %)			
Family atopy				
Yes	114 (51.6 %)	0.001	1.47 (1.02–2.10)	0.03
No	143 (37.7 %)			
Atopic dermatitis				
Yes	29 (56.9 %)	0.03	1.52 (0.81–2.86)	0.18
No	228 (41.5 %)			
Carpet at home				
Yes	194 (44.7 %)	0.13	1.21 (0.82–1.79)	0.33
No	63 (38 %)			
Cat				
Yes	78 (49.7 %)	0.04	1.41 (0.96–2.09)	0.07
No	179 (40.4 %)			
Dog				
Yes	74 (43 %)	0.95	–	–
No	183 (42.8 %)			
Moulds				
Yes	159 (45.2 %)	0.16	0.98 (0.68–1.41)	0.94
No	98 (39.5 %)			
Allergic rhinitis symptoms				
Yes	174 (57.8 %)	<0.001	3.12 (2.19–4.42)	<0.001
No	83 (27.8 %)			
Wheezing during the last 12 months				
Yes	34 (68 %)	<0.001	2.43 (1.27–4.66)	0.007
No	223 (40.5 %)			

## Discussion

This is the first study reporting on the pattern of allergic sensitization on a sample of young adults in sub-Saharan Africa. The prevalence of sensitization to common aeroallergens in this study was 41.8 %. Dust mites and cockroach were the most frequent allergens. Monosensitization was observed in 13.2 % of the sample and cockroach was the most implicated allergen. We also found an independent association between positive skin prick tests and family atopy, allergic rhinitis symptoms and asthma symptoms.

Many studies have been conducted worldwide showing a high prevalence of positive skin prick tests to common aeroallergens in the general population. In Europe, the Global Allergy and Asthma European Network survey showed that this prevalence varied from 31.4 to 52.9 % [12] while in the United States, a similar trend was observed in 2 national surveys, with almost half of the population aged 6 years and over having at least one positive skin prick test to common allergens [7, 13]. Another study in Vietnam found that one-third of the population was sensitized to common allergens [14]. In Africa, we found one study in Tunisia, showing a prevalence of 34 %. In sub-Saharan Africa, studies in general population are scarce. In Cameroon, a recent study showed that 32 % of a population of individuals with no allergic symptoms had one or more positive skin prick test to common aeroallergens [15].

In our study population, mites were the most common allergens. This is consistent with previous reports from different regions of the world [5, 7, 9, 12, 15]. Similar results were also found in asthma patients in sub-Saharan Africa [16, 17]. Cockroach was the fourth most frequent allergen in our study after the 3 mites. This finding corroborates previous studies in sub-Saharan Africa [15, 17]. The high prevalence of sensitization to both mites and cockroaches in this study is explained by the high relative humidity and hot climate observed in the city of Douala. In western countries, there is a geographical variation of the prevalence of sensitization to aeroallergens, with cockroach sensitization generally less frequent [7, 12, 18]. However, some studies conducted in Eastern Europe showed a high prevalence of cockroach sensitization [19, 20].

Sensitization to the other allergens such as moulds, as well as cat and dog dander had relative low prevalence in this study. Conversely, in European and North American countries, sensitization to cat and dog dander have a more important place among common aeroallergens [7, 18]. In these countries, the two pets are present more frequently in homes than they are in subSaharan Africa.

The independent association between sensitization to common aeroallergens and allergic rhinitis or asthma in the current study confirms previous findings [1]. Positive skin prick tests are considered as a hallmark of atopy. Indeed, it is well known that this genetic predisposition to develop IgE-mediated sensitivity to common aeroallergens is the strongest identifiable predisposing factor to the development of allergic rhinitis and asthma [21]. In sub-Saharan Africa, many studies had demonstrated the association between sensitization to mites and asthma. These mites are the main contributor to sensitization to common aeroallergens [22–24]. Having a family history of atopy was associated to sensitization to common aeroallergens. This finding which is in line with the results of Pallasaho et al. in Finland [25] can be explained by the genetic factors that influence the expression of atopy. In fact, allergy and organ-based phenotypes have strong heritability, but the exact genes involved in the expression of different disease phenotypes are only just being revealed and studies are still ongoing [26].

We did not find any association between sensitization to commons allergens and age. This is probably due to the fact that our participants were all young adults, with a mean age of 22.7 years. We could not compare them with a younger or older population. Usually, the rate of allergic sensitization decreases with increasing age [25, 27].

This study has several limitations: first the measurement of serum specific IgE to help confirm sensitization was not performed in this study. Secondly, we did not perform skin prick tests to pollens. This was because the commercial extracts available are adapted to the vegetation of western countries which are not necessarily present in sub-Saharan Africa. Another limitation of this study is the fact that our results cannot be generalizable to the whole Cameroon population because we included a selected population of university students in this study.

## Conclusion

This study revealed that a significant proportion of young adults in Cameroon are sensitized to common aeroallergens with dust mites identified as the most prevalent allergens. In order to recommend proper allergen avoidance and to prescribe allergen specific immunotherapy, skin prick tests containing mites and cockroach allergens should be considered in patients presenting with symptoms of asthma and allergic rhinitis.
